# Effect of *Cordyceps militaris* Residue and *Lactiplantibacillus plantarum* on Fermentation Quality and Bacterial Community of Alfalfa Silage

**DOI:** 10.3390/microorganisms13081919

**Published:** 2025-08-17

**Authors:** Luheng Wei, Meirong Zhao, Jia Song, Duo Gao, Xinnan Li, Juanjuan Sun, Zhu Yu, Chunsheng Bai

**Affiliations:** 1 College of Horticulture, Shenyang Agricultural University, Shenyang 110866, China; wlh20011016@163.com (L.W.); zhaomr365@163.com (M.Z.); 15042303167@163.com (J.S.); 2 Liaoning Inspection, Examination and Certification Center, Shenyang 110016, China; goddard518@126.com (D.G.); lixinnanli@163.com (X.L.); 3 Institute of Grassland Research, Chinese Academy of Agricultural Sciences, Hohhot 010010, China; sjj8234@126.com; 4 College of Grassland Science and Technology, China Agricultural University, Beijing 100193, China; yuzhu33150@sina.com

**Keywords:** *Cordyceps militaris* residue, alfalfa silage, NH_3_-N, random forest analysis

## Abstract

In order to reduce the environmental burden associated with *Cordyceps militaris* residue, we conducted a study to explore the feasibility of *Cordyceps militaris* residue as a silage additive. The experimental treatments included distilled sterile water control (CK), *Lactiplantibacillus plantarum* (LP, 1 × 10^5^ cfu/g), *Cordyceps militaris* residue (CM, 4% of fresh matter), and their combination (LP + CM). A total of 48 samples (4 treatments × 4 storage periods × 3 replicates) were prepared for the analysis of fermentation quality, chemical composition, microbial population, and bacterial community composition at days 2, 7, 28, and 45 of ensiling. Results showed that compared with the control, the CM and LP + CM treatments increased the lactic acid bacteria number and lactic acid content (*p* < 0.05), and a decrease in pH value and NH_3_-N content was observed (*p* < 0.05). The bacterial diversity in the CM group was higher and lower than that in the CK group in the early and late stages of fermentation, respectively, and maintained a higher relative abundance of *Lactococcus* on day 7. *Lactobacillus* remained the predominant bacterial community at the end of fermentation. Random forest analysis indicated that *Lactobacillus* was a key determinant of the pH, lactic acid, and acetic acid levels. Consequently, the addition of *Cordyceps militaris* residue alone, or combined with *Lactiplantibacillus plantarum*, improved the quality of alfalfa silage by increasing lactic acid and lowering NH_3_-N, providing a new approach for its development and utilization.

## 1. Introduction

*Cordyceps militaris* is a common medicinal fungus belonging to the phylum *Ascomycota* [1]. *C. militaris* contains several active ingredients, which are used to produce medicines and health products. Nowadays, *C. militaris* has gained growing recognition as a potential alternative to *Cordyceps sinensis* due to its comparable chemical capabilities and medicinal properties [2]. According to the market analysis report, global *C. sinensis* and *C. militaris* market was estimated to be valued at USD 1072.5 million in 2022 and was predicted to grow by 10.9% CAGR (compound annual growth rate) from 2022 to 2030 [3]. However, the expansion of artificial *C. militaris* cultivation has led to generate a large amount of solid medium residues and mycelial waste [4]. These residues are classified as waste and managed through conventional disposal methods such as incineration and landfilling, both of which lead to significant environmental concerns through either direct pollution or long-term ecological impacts [5,6,7,8]. Research has shown that many active substances remain in the residues after the processing of traditional Chinese medicines, and these can be repurposed as silage additives to enhance the fermentation quality and increase the feed value [9]. The application of herbal residues, astragalus residues, *Rosa roxburghii* pomace, and sea buckthorn pomace improved the fermentation quality of alfalfa (*Medicago sativa* L.) silage by means of favoring lactic acid bacteria activity and suppressing undesirable microorganisms [10,11,12,13]. Research indicates that *C. militaris* boasts various bioactive ingredients, including cordycepin, cordyceps polysaccharides, cordycepin acid, and sterols [14,15,16], which makes its residue suitable for development into additives. The development and utilization of *C. militaris* residue as a silage additive demonstrate significant environmental and economic benefits.

Alfalfa is widely used to feed livestock as a forage with high protein and abundant vitamins and minerals that animals need. However, its low dry matter and water-soluble carbohydrate contents, combined with a strong buffering capacity, make it notoriously challenging to ensile. During the fermentation of alfalfa silage, lactic acid bacteria are often unable to competitively dominate the microbial community and inhibit the reproduction of undesirable microorganisms, which can result in the failure of alfalfa ensiling [17]. Lactic acid bacteria has been applied in the production of silage, in particular, *Lactiplantibacillus plantarum* (formerly *Lactobacillus plantarum*) [18]. Guo et al. [19] found that inoculating with *L. plantarum* could alter the microbial composition and succession of alfalfa silage. Yang et al. [20] demonstrated that inoculating alfalfa silage with *L. plantarum* could enhance lactic acid bacteria proliferation, enabling them to rapidly dominate the bacterial community. *L. plantarum* inoculation in alfalfa silage significantly enhanced lactic acid bacteria dominance while effectively suppressing the growth and reproduction of *Enterobacteria* [21]. Taken together, the inoculation of *L. plantarum* inhibits the activity of putrefying bacteria and ensures the proliferation and competitive advantage of lactic acid bacteria, thus playing an integral role in successful alfalfa silage. Meanwhile, the bioactive compound cordycepin, present in *C. militaris* residue, can inhibit the growth of undesirable microorganisms such as *Bacillus subtilis*, *Escherichia coli*, and *Staphylococcus aureus* [22,23,24]. Drawing from these findings, we hypothesized that including *C. militaris* residue in alfalfa at ensiling could yield favorable impacts on the proliferation of lactic acid bacteria and help thwart the reproduction of harmful microorganisms. To our knowledge, no research on using *C. militaris* residue as a silage additive has previously been reported. Therefore, to access the impact of *C. militaris* residue, *L. plantarum*, and their combinations as silage additives on the fermentation property and bacterial community in alfalfa silage.

## 2. Materials and Methods

### 2.1. Silage Preparation

Alfalfa (Juneng No. 2) planted in an approximately 300 m^2^ experimental field in the Baicaoyuan Research Station (41°50′ N, 123°34′ E) of Shenyang Agricultural University (Liaoning, China) was managed with fertilization and irrigation and harvested through manually cutting at a location approximately 5 cm above ground at 10:00 a.m. Using a commercial grass chopper (Donghong No. 1, Donghong Mechanical Equipment Co., Ltd., Zhengzhou, China), the harvested alfalfa was processed into 1–2 cm segments. The experimental setup included four treatment groups: (i) sterile water control (CK); (ii) *Lactiplantibacillus plantarum* (LP, 1 × 10^5^ cfu/g of fresh matter (FM)); (iii) *C. militaris* residues (CM, 4% of FM, Shenyang Ivy Biotechnology Co., Ltd.,. Shenyang, China); and (iv) *Lactiplantibacillus plantarum* and *C. militaris* residue (LP + CM). The sterile water containing additives was evenly sprayed onto the materials. Then, the mixed forage was manually packed into polyethylene bags (20 cm × 30 cm) and vacuum-sealed. There were 12 bags for each treatment, among which triplicate bags were opened and sampled after 2, 7, 28, and 45 days of ensiling, respectively. A total of 48 samples (4 treatments × 4 storage periods × 3 replicates) were prepared for analysis of fermentation quality, chemical composition, microbial population, and bacterial community composition.

### 2.2. Fermentation Quality and Nutrient Composition Analyses

To determine the dry matter (DM) content, fresh and ensiled samples were measured following a 48-h drying period at 65 °C. The determination of neutral detergent fiber (NDF) content and acid detergent fiber (ADF) content adhered to the method of Van Soest et al. [25], utilizing an ANKOM A200i fiber analyzer (ANKOM Technology Corp., Fairport, NY, USA). For the total nitrogen (TN) content, the Kjeldahl method [26] was followed, utilizing an autoanalyzer (Kjeltec 8400; FOSS Co., Ltd., Hillerød, Denmark). The total nitrogen (TN) values were subsequently multiplied by 6.25 to determine the crude protein (CP) content. Water-soluble carbohydrate (WSC) content was determined using the colorimetric method after fully reacting with anthrone reagents [27].

A 20-g sample of silage was combined with 180 mL of sterile distilled water and then refrigerated at 4 °C for 12 h. Following this, the pH of the resulting extract was measured with a PB-10 pH meter (Sartorius Group, Goettingen, Germany) [28]. Using high-performance liquid chromatography, the organic acid concentrations were analyzed, in accordance with the method of Bai et al. [29]. The ammonia nitrogen (NH_3_-N) levels were measured via colorimetry using the phenol–sodium hypochlorite method [30]. A 10-g sample of silage was blended with 90 mL of distilled water, agitated at 180 rpm for 30 min, and then serially diluted. Yeasts and lactic acid bacteria were counted by placing them on rose Bengal medium and MRS agar (Beijing Aoboxing Bio-tech Co., Ltd., Beijing, China) [31].

### 2.3. Bacterial Community Analysis

Subsamples (10 g) of silage samples were shaken well with 90 mL of sterile distilled water using a cryogenic oscillator (THZ-98C, Shanghai Yiheng Scientific Instrument Co., Ltd., Shanghai, China), maintained at 4 °C and 180 rpm, for a duration of 30 min. The mixtures were then strained through quadruple-layered sterile gauze. The resulting filtrate underwent centrifugation at 8000 rpm for 15 min in a refrigerated centrifuge (ST 16R, Thermo Fisher Scientific, Inc., Waltham, MA, USA) maintained at 4 °C to concentrate the sediment. These sediments were then dispatched for high-throughput sequencing analysis [32].

Following the guidelines provided by the manufacturer, the E.Z.N.A.^®^ soil DNA Kit (Omega Bio-tek, Norcross, GA, USA) was utilized to isolate DNA from both the alfalfa and silage specimens. A polymerase chain reaction (PCR) was then conducted to amplify the V3–V4 sections of the bacterial 16S rRNA gene using tailored forward primer 341F (5′-CCTAYGGGRBGCASCAG-3′) and reverse primer 806R (5′-GGACTACNNGGGTATCTAAT-3′). The PCR product was retrieved from a 2% agarose gel, subjected to purification with the PCR Clean-Up Kit (YuHua, Shanghai, China), and subsequently quantified using a Qubit 4.0 fluorometer (Thermo Fisher Scientific, USA). The purified PCR products underwent sequencing on the Illumina Nextseq2000 platform (Illumina, San Diego, CA, USA). Quality checking of the paired-end raw sequencing data was conducted using fastp, followed by sequence assembly with FLASH (FLASH 1.2.11). Subsequently, UPARSE (UPARSE 7.1) was employed to cluster sequences at 97% similarity into operational taxonomic units (OTUs). Alpha diversity and beta diversity metrics were employed to assess the microbial diversity and community structure variations across samples. The resemblance between microbial populations in various samples was evaluated using principal coordinate analysis (PCoA). To identify significant variations in species composition across multiple sample groups, the Kruskal–Wallis H test was performed. R studio was used to analyze the OTU values of each sample genus level, and then AI software (version 26.0.1) was used to build the random forest model. The sequencing data have been deposited into the NCBI Sequence Read Archive database (accession: PRJNA750590).

### 2.4. Statistical Analysis

The experimental data were tested for normality and homogeneity of variance using SPSS (SPSS 22.0 program, SPSS Inc., Chicago, IL, USA), meeting the prerequisite requirements of normal distribution and homogeneity of variance for the two-way analysis of variance (ANOVA).The effects of additives, ensiling duration, and their interaction on fermentation quality, microbial counts, and bacterial community indices of silage were analyzed using two-way ANOVA in SPSS. The statistical model was *Y_ıjh_* = *µ* + *α_ι_* + *β_j_* + *αβ_ıj_* + ε*_ıjh_*, where *Y_ıjh_* is an observation, *µ* is the overall mean, *α_ι_* is the effect of the additives (*ı* = CK, LP, CM, LP + CM), *β_j_* is the number of ensiling days (*j* = 2, 7, 28, 45), *αβ_ıj_* is the additives × the number of ensiling days interaction, and ε*_ıjh_* is the error. Significant differences were determined using Duncan multiple range tests, with *p* < 0.05 considered statistically significant.

## 3. Results

### 3.1. Chemical and Microbial Characteristics of Alfalfa and Cordyceps militaris Residue Before Ensiling

The DM and CP contents were notably higher in the *C. militaris* residue compared with fresh alfalfa (*p* < 0.05) (Table 1). Conversely, the NDF and ADF content in *C. militaris* residue was significantly lower than that in fresh alfalfa (*p* < 0.05). *C. militaris* residue exhibited lower yeast counts compared with fresh alfalfa (*p* < 0.05).

### 3.2. Fermentation Quality of Alfalfa Silage

During the second day of ensiling, the pH value was lower in the CM and LP + CM groups than that in the CK group (*p* < 0.05) (Table 2). The alfalfa silage treated with LP + CM showed a marked increase in lactic acid concentration over the CK group during the ensiling process (*p* < 0.05). After 7d of ensiling, the CM and LP + CM groups exhibited a significantly lower acetic acid content compared with the CK group (*p* < 0.05). After 28 days of ensiling, the NH_3_-N concentration in both the CM and LP + CM groups was substantially lower than in the CK group (*p* < 0.05). At 45 days of ensiling, the LP, CM, and LP + CM silages all demonstrated substantially greater lactic acid bacterial counts than the CK group (*p* < 0.05).

### 3.3. Chemical Composition of Alfalfa Silage

The DM levels were notably greater in the CM and LP + CM treatments compared with the CK and LP groups (*p* < 0.05) (Table 3). The CP content in the CM and LP + CM treatments substantially exceeded that in the CK group (*p* < 0.05). The WSC content showed a marked increase in the CM and LP + CM groups relative to both CK and LP (*p* < 0.05).

### 3.4. The Microbial Community of Alfalfa Silage During Ensiling

The Chao index was noticeably higher in the CM group compared with the CK group at day 2 of ensiling (*p* < 0.05) (Table 4). By day 7 of ensiling, the Shannon index was significantly greater in the CM and LP + CM groups compared with CK (*p* < 0.05). With the extension of ensiling time, the alpha diversity of all treatment groups showed a decreasing trend.

**Figure 1:**
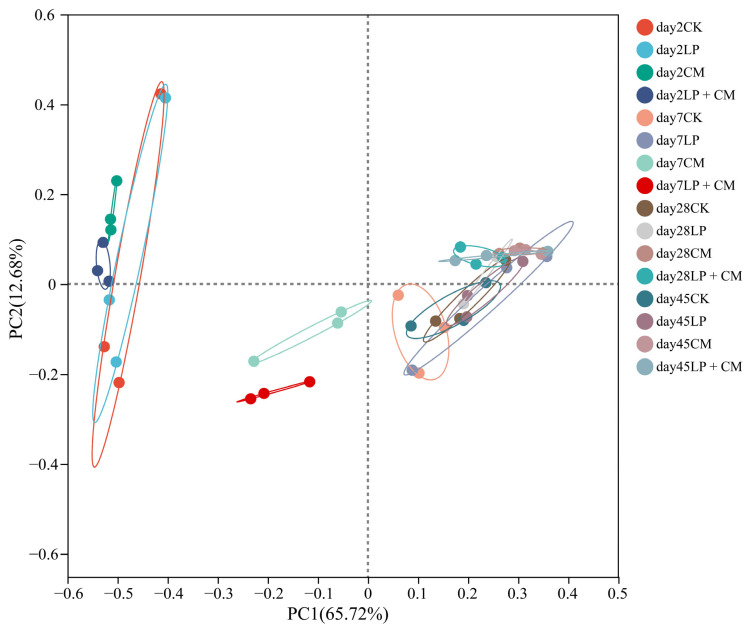
Principal coordinate analysis of the bacterial community in alfalfa silage after ensiling for 2, 7, 28 and 45 days (CK, control; LP, *L. plantarum*; CM, *Cordyceps militaris* residue; LP + CM, *L. plantarum* and *Cordyceps militaris* residue).

The principal coordinate analysis of the alfalfa silage microbial communities at different fermentation stages showed that PC1 contributed 65.72% of the total variance, and PC2 explained 12.68% of the total variance (Figure 1). The CM and LP + CM microbial communities showed clear separation from CK within the initial 7 days. As the silage duration increased, microbial populations within each treatment largely stabilized.

**Figure 2:**
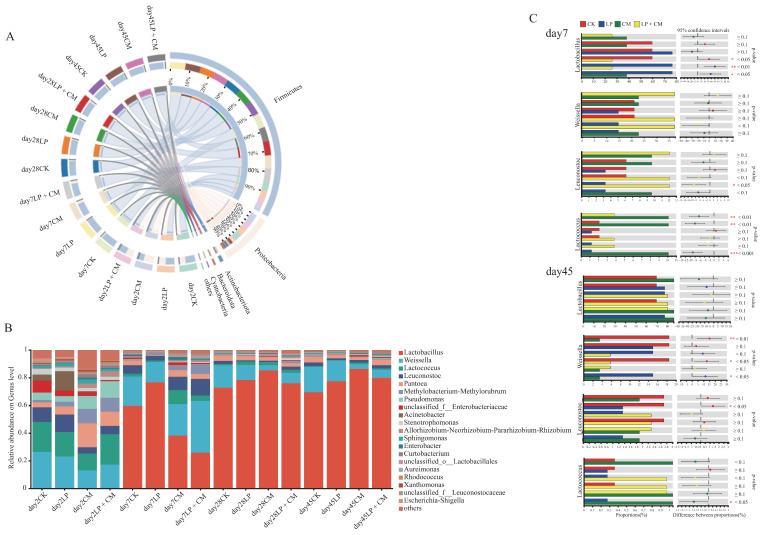
Changes in the relative abundances of bacteria at the phylum level (**A**), genus level (**B**), and the significance of intergroup differences (**C**) in alfalfa silage after ensiling for 2, 7, 28, and 45 days (CK, control; LP, *L. plantarum*; CM, *Cordyceps militaris* residue; LP + CM, *L. plantarum* and *Cordyceps militaris* residue).

By the second day of ensiling, the dominant phyla across all treatment groups were *Firmicutes* and *Proteobacteria*. After a week of fermentation, *Firmicutes* emerged as the predominant phylum within the microbial population (Figure 2A). By day 2 of the ensiling process, the predominant bacterial genera were primarily consisted of *Weissella* and *Lactococcus*. After seven days of fermentation, *Lactobacillus* and *Weissella* dominated all treatment groups. Following 28 days of ensiling, *Lactobacillus* emerged as the predominant bacterial community across all treatment groups (Figure 2B). On day 7 of ensiling, the LP + CM group showed a marked decline in *Lactobacillus* abundance compared with the CK group (*p* < 0.05) (Figure 2C). The CM group had a substantially greater abundance of *Lactococcus* than the other groups (*p* < 0.05). At 45 days of ensiling, the *Weissella* abundance in both the CM and LP + CM treatments was significantly reduced relative to the CK group (*p* < 0.05).

**Figure 3:**
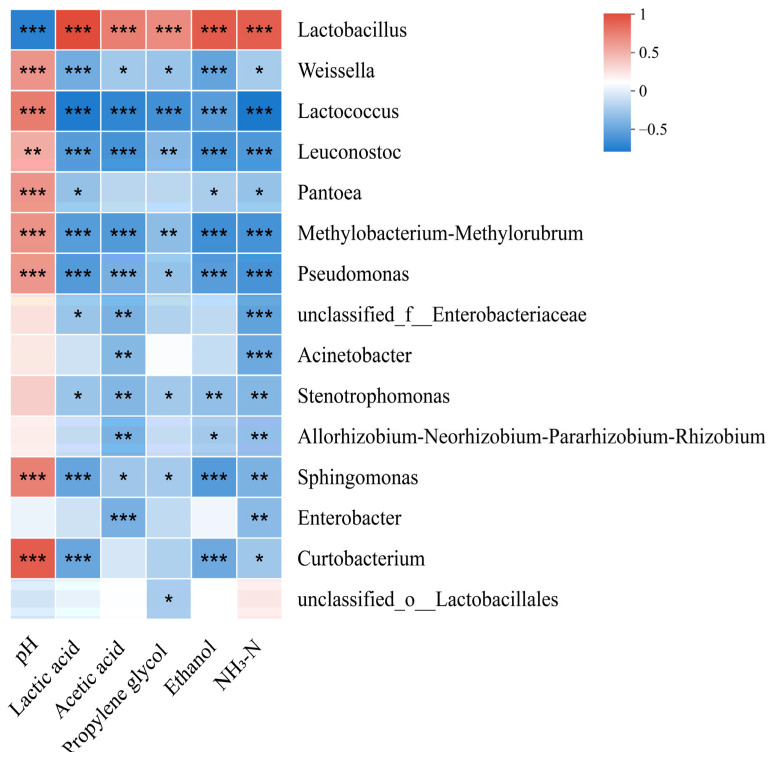
Correlation of the bacterial community and silage fermentation (NH_3_-N, ammonia nitrogen; *p*-values are shown as follows: * *p* < 0.05; ** *p* < 0.01; *** *p* < 0.001).

The pH levels exhibited an inverse relationship with *Lactobacillus* abundance, whereas they were positively associated with the abundances of *Weissella*, *Lactococcus*, and *Leuconostoc* (*p* < 0.05) (Figure 3). However, lactic acid, acetic acid, propylene glycol, ethanol, and NH_3_-N contents demonstrated opposite trends.

**Figure 4:**
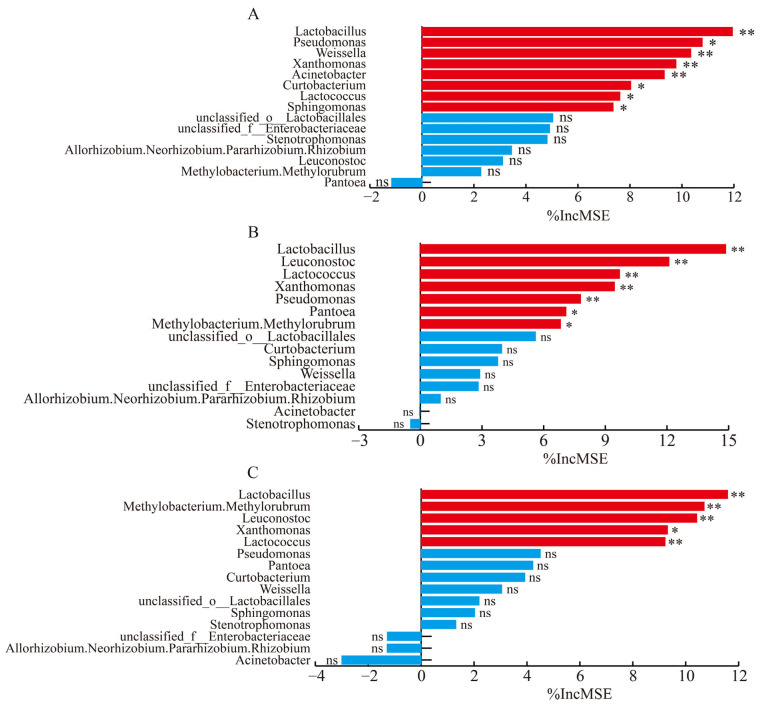
The importance of microorganisms in the random forest model for pH (**A**), lactic acid (**B**), and acetic acid (**C**). Red bars indicate statistically significant contributions (*p* < 0.05), while blue bars indicate no statistically significant contributions (*p* > 0.05). Levels of significance are shown as follows: ns, no significance; * *p* < 0.05; ** *p* < 0.01.

*Lactobacillus*, *Weissella*, *Xanthomonas*, *Acinetobacter*, etc. were the primary microorganisms influencing pH (Figure 4A). The main microorganisms affecting lactic acid content were *Lactobacillus*, *Leuconostoc*, *Lactococcus*, *Xanthomonas,* and others (Figure 4B). *Lactobacillus*, *Leuconostoc*, *Xanthomonas*, and *Lactococcus* had the greatest impact on the acetic acid content (Figure 4C). Among these, *Lactobacillus* had a significant impact on the pH level as well as on the concentrations of lactic acid and acetic acid.

## 4. Discussion

### 4.1. The Characteristics of Raw Material

The DM content of raw material prior to ensiling plays a pivotal role in determining the silage quality [33]. The DM content in alfalfa fell below the ideal range (30–40%) for silage preparation. The WSC content of alfalfa and *C. militaris* residue was 8.76 and 6.90% DM, falling within the recommended range for ensuring silage quality [34]. The lactic acid bacteria population in fresh alfalfa exceeded 10^5^ cfu/g, ensuring the successful ensiling [31,32,33,34,35]. The amount of yeast in the *C. militaris* residue was lower than that in alfalfa, which is related to a high DM content. The higher DM content can effectively suppress the proliferation of undesirable microorganisms [36].

### 4.2. Fermentation Quality and Chemical Composition of Alfalfa Silage

The CM and LP + CM groups exhibited a lower pH level compared with the CK group, demonstrating that *C. militaris* residue supplementation can effectively enhance alfalfa silage fermentation. This outcome may be due to the increased number of lactic acid bacteria after adding *C. militaris* residue, thereby producing more lactic acid, ultimately lowering the pH [10].Moreover, the LP treatment group exhibited pH levels comparable to the control group, and this pH consistency was maintained throughout the ensiling process. This finding aligns with Ni et al. [13], suggesting that overly high moisture in alfalfa could hinder the pH-modulating effect of exogenous additives.

The pH decline in alfalfa silage results from the accumulation of fermentation acids, primarily lactic acid. Lactic acid is mainly produced by lactic acid bacteria consuming WSC [37]. In this study, the addition of *C. militaris* residue increased the lactic acid content compared with the CK group. That result was similar with the previous report, in which the bioactive elements in sea buckthorn pomace could boost the metabolic prowess of *L. plantarum*, which in turn results in greater lactic acid production in the silage [38]. Some bioactive components in *C. militaris* residue might also elicit comparable effects. Acetic acid is a product of hetero-fermentation during the ensiling process [39]. Research indicates that a high WSC content in silage could can slow the transition from homofermentative to heterofermentative processes [40]. In this study, the CM and LP + CM treatments demonstrated a notably greater WSC content than the CK group, which is the reason for the decrease in acetic acid levels observed in the CM and LP + CM groups compared with the CK group. NH_3_-N mainly results from the breakdown of CP caused by undesirable microorganisms [41]. After a 28-day period of silage, the NH_3_-N levels in both the CM and LP + CM treatments were significantly lower compared with the CK group, suggesting that adding *C. militaris* residue could effectively reduce protein degradation. This effect results from the lower pH levels suppressing harmful microbe proliferation, which in turn decreases its degradation of CP [42]. After 2 days of ensiling, the yeast counts in each treatment group rapidly decreased, which was due to acetic acid inhibiting the growth of yeast [43].

During the ensiling process, the main reason for DM loss during silage is the growth of harmful microorganisms including yeasts, molds, and Brucella [44]. Higher DM retention in alfalfa silage for the CM and LP + CM groups, as opposed to CK and LP, suggest that CM and LP + CM helped minimize DM loss during fermentation. Li et al. [45] also observed comparable results, noting that the incorporation of herbal residues inhibited harmful microbe proliferation, leading to decreased DM losses. In addition, the high dry matter content in *C. militaris* residue could also contribute to this result. Lee’s study [46] demonstrated that native proteolytic enzymes undergo denaturation under acidic conditions (pH < 4.5), thereby effectively suppressing protein degradation and improving the silage fermentation quality. This research provides a plausible explanation for the higher CP content in CM and LP + CM groups compared with the CK group. In this study, the water-soluble carbohydrate content in the alfalfa silage samples from the CM and LP + CM groups was higher when compared with those from the CK and LP groups. This striking difference could be explained by the swift drop in pH levels, which effectively curbed the proliferation of harmful microorganisms. This suppression, in turn, limited their consumption of WSC, leading to the conservation of a greater amount of WSC. Chen et al. [10] also observed comparable results, noting that sea buckthorn pomace supplementation effectively preserved more WSC by restricting WSC utilization by undesirable microbial populations.

### 4.3. The Microbial Community of Alfalfa Silage

Each group showed near-complete coverage (above 0.99), demonstrating that our results accurately depicted the sampled biological community [47]. In the initial phase of ensiling, the Shannon and Chao diversity indices were elevated in the CM and LP + CM groups compared with the CK group. This result was similar to Wu et al. [48], who reported that adding *N. cadamba* leaf meal to stylo silage could increase the bacterial diversity in the initial phase of ensiling. As the duration of ensiling increased, the alpha diversity of all treatment groups showed a decreasing trend, which may be due to the gradual dominance of lactic acid bacteria replacing the originally diverse microbial population in the silage [17]. Early on in the ensiling process, the CM and LP + CM groups exhibited a separation from the CK group, indicating a shift in the composition of microbial communities. As the silage period progressed, the microbial communities in each group started to converge. One possible reason is that as the pH declined across all treatment groups, *Lactobacillus* gradually dominated the bacterial community, resulting in convergent microbial compositions among treatments [49].

The main bacteria found in alfalfa silage are *Firmicutes* and *Proteobacteria* [50]. In this study, *Firmicutes* and *Proteobacteria* initially dominated the microbial population during the early fermentation phase. As the fermentation process continued, *Firmicutes* emerged as the predominant phylum within the microbial community. This is related to the anaerobic and acidic conditions developed during silage, which can promote the growth of *Firmicutes* [51,52]. During the initial phase of ensiling, *Weissella* and *Lactococcus* participates in the fermentation process, but as the pH level drops in silage, their proliferation slows, leading to their eventual replacement by more acid-tolerant lactic acid bacteria [53]. Our study yielded comparable findings, demonstrating that the dominant bacterial genera primarily consisted of *Weissella* and *Lactococcus* at day 2 of ensiling, whereas *Lactobacillus* had taken over as the dominant genus after one week of ensiling. The CM treatment group showed a markedly higher abundance of *Lactococcus* compared with the other treatments at 7 days of ensiling. This revealed that *C. militaris* residue supplementation in alfalfa silage effectively maintained a higher relative abundance of *Lactococcus*. Peng et al. [42] reported similar findings, who observed that the addition of sea buckthorn pomace could preserve a higher population of *Lactococcus* during the initial stages of the silage process. Cai et al. [35] carried out a study showing that coccoid lactic acid bacteria (primarily *Lactococcus*, *Leuconostoc*, and *Weissella*) exhibit vigorous growth during the early fermentation stage. At day 7 of ensiling, the dominant bacteria in the LP + CM group was *Weissella*, indicating that it was in the early stage of fermentation. This is the reason why the abundance of *Lactobacillus* in the LP + CM group was lower than that in the CK group (*p* < 0.05). This result indicates that incorporating the *C. militaris* residue did not facilitate *Lactobacillus* in quickly becoming the dominant species within the bacterial community, aligning with the earlier observations made by Chen et al. [10]. Based on this analysis, the addition of *C. militaris* residue boosted bacterial diversity early in the silage process. However, it could not cause *Lactobacillus* to rapidly become the dominant strain, thereby prolonging the transition of the lactic acid bacteria from acid-intolerant to acid-tolerant types. Following 28 days of ensiling, the pH reduction inhibited the growth of *Weissella*, leading to its eventual replacement by acid-resistant *Lactobacillus* late in silage [54]. At the 45-day mark of the silage process, the *Weissella* abundance in the CM and LP + CM groups showed a significant decrease compared with the CK group. There were no significant differences in the abundance of *Weissella* between the LP and CK groups. This suppression may stem from reduced pH in the CM and LP + CM groups, which further restricts the growth of *Weissella* [43].

The pH value was inversely related to *Lactobacillus* abundance but positively correlated the abundances of *Weissella*, *Lactococcus*, and *Leuconostoc*. Lactic acid and acetic acid demonstrated opposite trends. *Weissella* and *Lactococcus* have poor tolerance to acidic environments and cannot survive when the pH decreases [53]. *Lactobacillus* plays a pivotal role in the production of lactic acid within alfalfa silage, ultimately driving pH reduction [55,56]. Thus, *Lactobacillus* is pivotal in lowering pH levels in the late phase of silage fermentation.

During the ensiling process, *Lactobacillus* gradually dominate the microbial community, promoting lactic acid accumulation and thus reducing the pH [57]. Due to the diversity of sugars in the fermentation substrate, acetic acid serves as another critical metabolic product of *Lactobacillus* fermentation besides lactic acid [17]. This is the reason why *Lactobacillus* served as pivotal determinants in influencing the pH level as well as the concentrations of lactic acid and acetic acid in the random forest analysis.

## 5. Conclusions

In comparison to the CK group, both the CM and LP + CM treatment groups exhibited enhanced lactic acid accumulation and reduced pH levels, indicating enhanced fermentation quality. The incorporation of *C. militaris* residue decreased the alpha diversity at the end phase of fermentation. *C. militaris* residue could preserve more *Lactococcus* by day 7 of the ensiling process and inhibit the growth of *Weissella* at 45 days of ensiling. *Lactobacillus* still dominated the bacterial community in the late stage of fermentation. Thus, *C. militaris* residue can be applied as a silage additive to enhance the fermentation quality of alfalfa silage, providing a new approach for its development and utilization. Nevertheless, to develop and ensure the use of *C. militaris* residue as a silage additive, it is necessary to further evaluate the effect of combining *C. militaris* residue with other microbial strains as well as the aerobic stability of the treated silage and its impact on animal performance.

## Figures and Tables

**Table 1:** Chemical and microbial characteristics of fresh alfalfa and *Cordyceps militaris* residue.

Item	Alfalfa	*Cordyceps militaris* Residue	*p*-Value
Dry matter (%FM)	21.33 ± 0.18	89.80 ± 0.09	<0.001
CP (%DM)	21.09 ± 0.32	22.53 ± 0.33	0.006
NDF (%DM)	37.42 ± 1.35	33.11 ± 0.92	0.010
ADF (%DM)	27.00 ± 1.20	17.19 ± 0.59	<0.001
WSC (%DM)	8.76 ± 1.77	6.90 ± 0.46	0.152
Lactic acid bacteria (log_10_ cfu/g)	5.40 ± 0.31	4.74 ± 0.12	0.026
Yeasts (log_10_ cfu/g)	5.82 ± 0.22	4.83 ± 0.11	0.002

DM, dry matter; CP, crude protein; NDF, neutral detergent fiber; ADF, acid detergent fiber; WSC, water-soluble carbohydrates; cfu, colony forming units.

**Table 2:** The effects of adding *Cordyceps militaris* residue on the fermentation quality of alfalfa silage during 45 days of ensiling.

Item	Treatment	Ensiling Days				SEM	*p*-Value		
		2	7	28	45		T	D	T*D
pH	CK	4.89 Aa	4.47 b	4.49 Ab	4.41 Bb	0.006	<0.001	<0.001	<0.001
	LP	4.91 Aa	4.39 c	4.46 Ab	4.49 Ab				
	CM	4.77 Ba	4.47 b	4.23 Bc	4.16 Cc				
	LP + CM	4.72 Ba	4.41 b	4.17 Bc	4.14 Cc				
Lactic acid (%DM)	CK	2.11 Bb	2.74 Bb	6.64 Ba	7.13 Ba	0.028	<0.001	<0.001	0.031
	LP	2.18 ABd	4.08 Ac	6.72 ABb	8.23 Aa				
	CM	2.27 Ad	3.69 Ac	7.00 ABb	8.45 Aa				
	LP + CM	2.25 Ad	3.72 Ac	7.06 Ab	8.19 Aa				
Acetic acid (%DM)	CK	1.02 b	1.87 Aa	2.41 Aa	2.31 Aa	0.040	<0.001	<0.001	0.132
	LP	1.47 b	1.43 ABb	2.25 Aa	2.51 Aa				
	CM	1.15 b	1.08 Bb	1.69 Ba	1.92 Ba				
	LP + CM	1.01 c	0.96 Bc	1.69 Bb	2.00 Ba				
1, 2 propylene glycol (%DM)	CK	0.00 b	0.00 b	0.00 b	0.26 Ba	0.005	<0.001	<0.001	<0.001
	LP	0.00 b	0.00 b	0.00 b	0.29 Aa				
	CM	0.00 c	0.00 c	0.01 b	0.04 Ca				
	LP + CM	0.00 b	0.00 b	0.01 b	0.05 Ca				
Ethanol (%DM)	CK	0.86 b	1.13 b	1.25 BCa	1.16 Ca	0.017	0.002	<0.001	0.412
	LP	0.92 c	1.08 b	1.32 Ba	1.35 ABa				
	CM	0.93 b	1.12 b	1.42 Aa	1.50 Aa				
	LP + CM	0.84 b	0.92 b	1.22 Ca	1.24B Ca				
NH_3_-N %TN	CK	0.57 c	2.14 b	4.75 Aa	5.01 Aa	0.034	<0.001	<0.001	<0.001
	LP	0.86 d	1.79 c	4.79 Aa	3.52 Cb				
	CM	0.72 d	1.50 c	2.97 Bb	4.02 Ba				
	LP + CM	0.82 c	1.50 b	2.84 Ba	3.05 Da				
Lactic acid bacteria, log_10_ cfu/g FM	CK	7.37 Bb	10.05 ABa	9.69 a	9.49 Ca	0.061	<0.001	<0.001	<0.001
	LP	7.92 Bb	9.42 Ba	9.79 a	9.68 ABa				
	CM	8.91 Ac	10.81 Aa	9.95 b	9.61 ABb				
	LP + CM	9.33 Ab	9.64 Bab	10.19 a	9.77 Aab				
Yeasts, log_10_ cfu/g FM	CK	5.67 a	3.22 b	3.10 b	1.80 c	-	-	-	-
	LP	5.34	<2.00	<2.00	3.73				
	CM	5.56	3.55	<2.00	3.82				
	LP + CM	5.55	3.18	3.92	<2.00				

DM, dry matter; NH_3_-N, ammonia nitrogen; TN, total nitrogen. CK, control; LP, L. plantarum; CM, *Cordyceps militaris* residue; LP + CM, *L. plantarum* and *Cordyceps militaris* residue. Different lower case letters indicate significant differences among different silage days under the same treatment, different upper case letters indicate significant differences among different treatments under the same silage day. SEM, standard error means; T, treatment; D, ensilage days; T*D, the interaction between treatments and ensiling days.

**Table 3:** The effects of adding *Cordyceps militaris* residue on the chemical composition of alfalfa silage after 45 days of ensiling.

Item	Treatments				SEM	*p*-Value
	CK	LP	CM	LP + CM		
DM (%FM)	21.69 c	22.13 c	24.84 a	23.99 b	0.404	<0.001
CP (% DM)	21.90 b	21.85 b	22.80 a	22.72 a	0.164	0.024
NDF (% DM)	33.93	34.90	33.90	34.33	0.345	0.770
ADF (%DM)	25.08	26.23	24.21	24.87	0.346	0.227
WSC (% DM)	1.35 c	1.20 c	2.10 a	1.71 b	0.372	<0.001

DM, dry matter; CP, crude protein; NDF, neutral detergent fiber; ADF, acid detergent fiber; WSC, water-soluble carbohydrates; CK, control; LP, *L. plantarum*; CM, *Cordyceps militaris* residue; LP + CM, *L. plantarum* and *Cordyceps militaris* residue. Means in the same row (a–c) with different lower case letters differ significantly from each other (*p* < 0.05). SEM, standard error means.

**Table 4:** Effect of additives and ensiling days on the bacterial alpha diversity of alfalfa silage during 45 days of ensiling.

Item	Treatment	Ensiling Days				SEM	*p*-Value		
		2	7	28	45		T	D	T*D
Shannon	CK	2.14 Ba	1.35 Bb	1.02 b	1.09 b	0.039	<0.001	<0.001	0.002
	LP	2.15 Ba	0.85 Bb	0.93 b	0.85 b				
	CM	2.91 Aa	1.96 Ab	0.79 c	0.76 c				
	LP + CM	2.55 ABa	1.94 Ab	1.17 c	0.99 c				
Chao	CK	175.15 B	147.15	113.81 B	147.28	4.571	0.039	<0.001	0.009
	LP	174.77 B	111.14	134.53 AB	117.40				
	CM	274.73 Aa	141.28 b	98.16 Bb	96.15 b				
	LP + CM	225.56 ABa	153.94 b	168.53 Aab	143.91 b				
Ace	CK	215.27	196.15	138.83 C	153.33 A	5.059	0.203	<0.001	0.003
	LP	187.87	127.29	173.40 B	148.05 A				
	CM	277.05 a	192.29 b	111.10 Dc	98.36 Bc				
	LP + CM	213.89	168.72	202.39 A	175.17 A				
Coverage	CK	0.99	0.99	0.99	0.99	0.000	0.245	<0.001	0.019
	LP	0.99	0.99	0.99	0.99				
	CM	0.99	0.99	0.99	0.99				
	LP + CM	0.99	0.99	0.99	0.99				

CK, control; LP, *L. plantarum*; CM, *Cordyceps militaris* residue; LP + CM, *L. plantarum* and *Cordyceps militaris* residue. Different lower case letters indicate significant differences among different silage days under the same treatment, different upper case letters indicate significant differences among different treatments under the same silage day. SEM, standard error means; T, treatment; D, ensilage days; T*D, the interaction between treatments and ensiling days.

## Data Availability

The original contributions presented in this study are included in the article. Further inquiries can be directed to the corresponding author.
